# Supporting adherence to adjuvant CDK4/6 inhibitors in women with early breast cancer (SWEET-PLUS): protocol for a multicentre UK study using qualitative and co-development methods

**DOI:** 10.1136/bmjopen-2026-119558

**Published:** 2026-08-11

**Authors:** Pearlin Teow, Lucy McGeagh, Henry Cain, Adam Todd, Farah Rehman, Jo Brett, Nicky Levitt, Mark Turner, Sarah-Jane F Stewart, Emma Hunt, Hazeline Terrado, Eila Watson, Linda Sharp

**Affiliations:** 1 Population Health Sciences Institute, Newcastle University, Newcastle upon Tyne, UK; 2 Oxford Institute of Applied Health Research, Oxford Brookes University, Oxford, UK; 3 Newcastle Upon Tyne Hospitals NHS Foundation Trust, Newcastle upon Tyne, UK; 4 School of Pharmacy, Newcastle University, Newcastle upon Tyne, UK; 5 Imperial College Healthcare NHS Trust, London, UK; 6 Oxford University Hospitals NHS Foundation Trust, Oxford, UK; 7 Research Software Engineering, Newcastle University, Newcastle upon Tyne, UK; 8 School of Psychology, University of Surrey, Guildford, UK

**Keywords:** Cancer, Medication Adherence, QUALITATIVE RESEARCH, Self-Management, Patient-Centered Care

## Abstract

**Abstract:**

**Introduction:**

Breast cancer is the most common cancer in women in the UK. For women who have early-stage oestrogen-receptor positive (ER+ve) disease, daily oral adjuvant endocrine therapy reduces risk of recurrence. Recently, CDK4/6 inhibitors, a form of biological therapy, have been approved for use alongside endocrine therapy. However, trial data suggest there may be challenges with adherence to CDK4/6 inhibitors. This study aims to explore the experiences of adherence and support needs of women who have been prescribed CDK4/6 inhibitors for early ER+ve breast cancer. It will also co-develop an intervention to support women with adherence to these drugs alongside endocrine therapy through an evidence-based, theory-informed and patient-centred approach.

**Methods and analysis:**

The SWEET-PLUS study has three phases. Phase I uses semi-structured interviews or focus groups to explore the experiences of women with early breast cancer who have been prescribed CDK4/6 inhibitors. It will also explore their support needs and experiences of adherence. Phase II involves interviews or focus groups with healthcare professionals who support CDK4/6 inhibitor prescription and associated care to understand the existing support and unmet needs. Phase I and II findings will inform phase III, which comprises workshops and user testing interviews to co-develop an intervention to support women with adherence to CDK4/6 inhibitors alongside endocrine therapy. This will be delivered as an additional module prototype designed for future integration into the existing HT&Me intervention, which supports women with adherence to endocrine therapy.

Data from phases I and II will be analysed using a framework approach-based thematic analysis. Phase III data will be analysed through content analysis.

**Ethics and dissemination:**

SWEET-PLUS received ethical approval from the National Health Services (NHS) Health Research Authority (Cambridge East Research Ethics Committee (25/EE/0220)). Research findings will be disseminated through peer-reviewed journal articles and via international and national conferences. Further dissemination will be guided by patient and public involvement.

**STRENGTHS AND LIMITATIONS OF THIS STUDY**

- Usage of a theory-informed framework that emphasises a no-blame approach towards medication adherence will enable comprehensive identification of the support needs of women on CDK4/6 inhibitors for early breast cancer.
- Recruitment through National Health Service sites and supplementary avenues such as social media, charities and support groups will create opportunities to engage with harder to reach populations, including those who have poorer breast cancer outcomes.
- Continual patient and public involvement throughout each phase will ensure that the study and the proposed intervention remain patient-centred.
- The developed prototype add-on module will be evidence-based, theory-informed, patient-centred and specifically designed for future integration into the existing HT&Me intervention, which is feasible and acceptable to women with breast cancer and health professionals for supporting adherence to endocrine therapy in women with early breast cancer.
- Participant recruitment will be restricted to women who choose to participate in the study and are able to speak and understand English due to funding limits.

## Introduction

 In the UK, almost 57 000 new breast cancers are diagnosed annually, and 11 200 women die from breast cancer.^1^ Most women are diagnosed at an early stage; 85% have stage I or II disease, which has not spread beyond local lymph nodes,^2^ and 7.5% have stage III disease,^1^ where the cancer has spread locally but not to distant organs. Breast cancer prognosis has improved since the 1990s,^3^ and survival rates for early-stage breast cancer are high. The 5-year net survival rate is 98.4, 90.3 and 75.6% for stage I, II and III disease, respectively.^4^ However, breast cancer can recur after surgery, radiotherapy and/or chemotherapy. Recurrence risk is highest for the initial years post-diagnosis but persists for more than 30 years.^5^ Approximately 80% of breast cancers are oestrogen sensitive (ER+ve).^1^ This means that the presence of oestrogen can stimulate the growth, or re-growth, of cancerous cells. Research has shown that up to 20% of ER+ve early breast cancers recur within 10 years.^5^ In addition, one-third of patients with stage 2 disease and one half of those with stage 3 will eventually recur.^6^ Among women and their families, fear of recurrence is a major unmet need.^7^

Women with early ER+ve disease are recommended daily adjuvant oral endocrine therapy for 5–10 years to reduce risks of recurrence and breast cancer death.^6^ Endocrine therapy works by blocking the effect of oestrogen or lowering the amount of oestrogen present in the body. The choice of medication (eg, tamoxifen or aromatase inhibitors such as letrozole, anastrozole and exemestane) is influenced by menopausal status, risk of recurrence and patient preferences.^8^ While endocrine therapies have been available for almost 50 years,^9^ there have been significant advances in pharmacological approaches to cancer treatment in the last decade. These approaches often target specific pathways of cancer pathogenesis (biological or precision therapies) or use the body’s immune system to target the cancer (immunotherapies). Until recently, these treatments were limited to advanced or metastatic cancer. However, in 2022, the National Institute for Health and Care Excellence (NICE) in England recommended a biological therapy—the CDK4/6 inhibitor abemaciclib—alongside adjuvant endocrine therapy in a subset of early ER+ve patients with breast cancer with higher recurrence risk (see table 1).^10^ In trials, abemaciclib tablets taken two times per day for 2 years (alongside endocrine therapy) reduced recurrence by 34% vs endocrine therapy alone.^11^ Another CDK4/6 inhibitor—ribociclib—has been found to benefit an even larger patient group (ER+ve, HER2-ve, stage 2/3, node negative/positive).^12^ Ribociclib has been under consideration by NICE for use in the early breast cancer setting and was given preliminary approval in April 2025.^13^

**Table 1:** Summary of features for the use of abemaciclib and ribociclib alongside endocrine therapy in the early-stage adjuvant breast cancer setting

Feature	Abemaciclib (TA810)	Ribociclib (TA1086)
Indication	Adjuvant with endocrine therapy	Adjuvant with aromatase inhibitor
Endocrine partner	Tamoxifen or aromatase inhibitor	Aromatase inhibitor (± ovarian suppression)
Subtype	HR positive, HER2 negative	HR positive, HER2 negative
Stage	Early breast cancer	Early breast cancer
Risk group	High risk (restricted)	High risk (broader)
Nodal status requirement	Node-positive only	Node-positive and some node-negative
Definition of high risk as per trial	≥4 nodes OR 1–3 nodes+tumour ≥5 cm or grade 3	Node-positive: ≥4 nodes OR 1–3 nodes + (≥5 cm OR grade 3)
		No nodes, but adverse tumour biology/size>5 cm OR 2–5 cm + (grade 3 OR high Ki‑67/genomic risk)
Schedule type	Continuous	Cyclical
To be taken	Two times per day	Taken once a day for 3 weeks on/1 week off
Duration of use	2 years	3 years

HR, hormone receptor.

However, non-adherence to CDK4/6 inhibitors (ie, intentional or unintentional failure to follow the recommended treatment regimen because of, for example, not starting the medication, deviating from the dosing schedule by skipping doses or taking reduced doses or stopping treatment early) may be a significant problem.^14–16^ In the adjuvant early-disease setting, 26% of trial participants discontinued abemaciclib, for reasons other than recurrence, before completing the recommended 2-year treatment.^17^ For ribociclib, only ~40% of trial participants completed the 3-year course.^12^ Since trial populations are often selected (ie, more homogeneous and less comorbid than the general clinical population with the condition), motivated and well supported, it seems very likely that adherence to CDK4/6 inhibitors could be even lower in the real-world setting of routine clinical practice.^18 19^ This phenomenon was observed in relation to adherence to endocrine therapy in breast cancer and many other medications.^20^

Medication non-adherence, while modifiable, is a complex behaviour with multiple determinants.^21^ A recent umbrella review of determinants of endocrine therapy non-adherence highlighted the influence of a wide range of inter-related factors.^22^ These include medication factors (eg, complexity of treatment regimen, side effects), patient-related factors (eg, available family/social support, beliefs about the cancer and medication) and healthcare system-related factors (eg, patient–professional relationship, follow-up care). Due to the contemporary nature of abemaciclib and ribociclib usage in the early breast cancer setting, there is lack of knowledge surrounding the determinants of CDK4/6 inhibitors non-adherence when used alongside endocrine therapy.

In recent years, there has been burgeoning interest in the development of interventions to support adherence to endocrine therapy.^23^ With the anticipated rapid uptake of CDK4/6 inhibitors alongside endocrine therapy,^24 25^ there is a pressing need for endocrine adherence-based interventions to incorporate CDK4/6 inhibitors into their design. This would ensure interventions adequately address any new or shifting support needs that may arise from the inclusion of CDK4/6 inhibitors alongside endocrine therapy.

We recently designed an evidence-based, theory informed, patient-centred self-management intervention—HT&Me—to support endocrine therapy adherence among women with ER+ve early breast cancer.^26^ HT&Me was developed within the SWEET (Improving Outcomes for Women diagnosed with early breast cancer through adherence to adjuvant endocrine therapy) research programme (https://www.sweetstudy.co.uk). It comprises (1) a short animation; (2) two personalised nurse/practitioner consultations (in-person or online); (3) an interactive web-app and (4) regular email or text reminders. It has been shown to be acceptable to women and healthcare professionals, and feasible to be delivered within the UK National Health Service; a large trial of effectiveness and cost-effectiveness is currently underway.^26–28^ The availability of HT&Me provides a foundation for the implementation of adherence support for CDK4/6 inhibitors in this same population.

## Aims and objectives

### Aim

SWEET-PLUS aims to explore the experiences of adherence and support needs of women who have been prescribed CDK4/6 inhibitors for early ER+ve breast cancer and to use these insights to inform the co-development of a prototype add-on module to the HT&Me intervention (online supplemental material A) to support women adhere to these drugs alongside endocrine therapy.

### Objectives

The objectives of SWEET-PLUS are to:

1. Explore the experiences of adherence and support needs of women who have been prescribed CDK4/6 inhibitors for early ER+ve breast cancer (*phase I*)
2. Investigate healthcare professionals’ experiences of prescribing CDK4/6 inhibitors in early breast cancer and providing associated follow-up care and views on patient support and unmet needs (*phase II*)
3. Co-develop, with patients, a prototype additional module for HT&Me to support women with adherence to CDK4/6 inhibitors (alongside endocrine therapy) (*phase III*)

## Methods and analysis

### Study status

SWEET-PLUS started on 1 August 2025 and obtained ethical approval on 20 October 2025. Phase I interviews started on 10 November 2025, Phase II interviews started on 6 January 2026. Phase I and II data collection is anticipated to end late summer 2026. All data collection is anticipated to end on 31 January 2027.

### Study design

**Figure 1:**
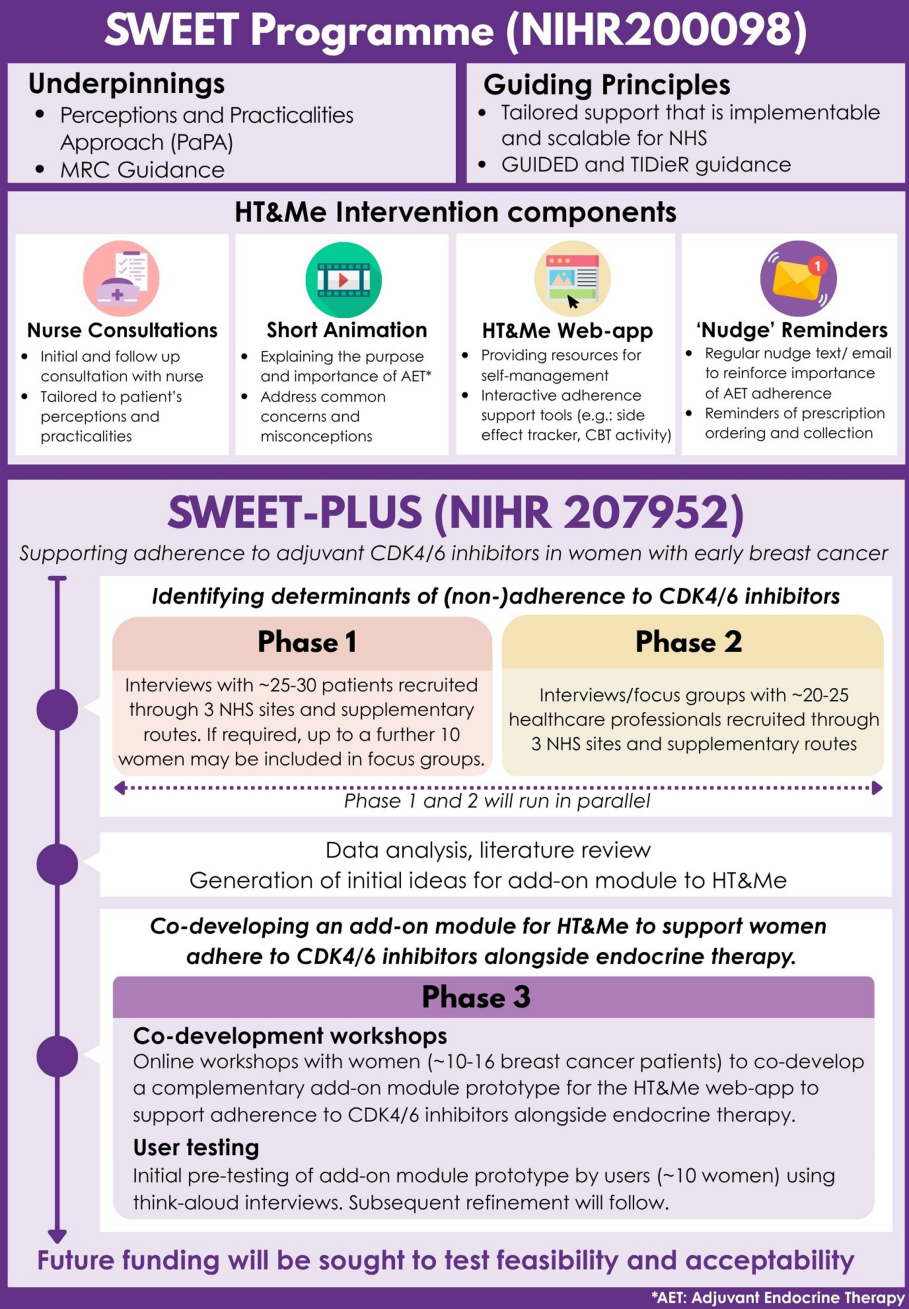
Overview of SWEET-PLUS and its relationship with the SWEET programme. CBT, cognitive behavioural therapy; GUIDED, GUIDance for the rEporting of intervention Development checklist; MRC, Medical Research Council; NHS, National Health Service; TIDieR, Template for Intervention Description and Replication.

The SWEET-PLUS study will adopt a qualitative approach incorporating semi-structured interviews, focus groups, online co-development workshops and user-testing interviews. The study has three phases. Phases I and II will run in parallel followed by phase III. The co-developed add-on module prototype will undergo user-testing and serve as an addition to the existing HT&Me intervention as part of the SWEET programme. Figure 1 provides an overview of SWEET-PLUS and its relationship with the SWEET programme.

### Theoretical framework

The Perceptions and Practicalities Approach (PaPA) will provide theoretical underpinnings for the study. The use of a theoretical framework can enable a comprehensive perspective through the integration of complex personal and contextual factors into the process of intervention development.^29 30^ Following the NICE Medicines Adherence Guidelines,^31^ PaPA adopts a ‘no-blame’ approach towards medicine non-adherence and suggests that support should be tailored to address specific perceptions (eg, beliefs about treatment and condition) and practicalities (eg, capabilities and resources) influencing motivation and ability to continue treatment.^32^ It specifies adherence support must take account of the patient’s evaluation of treatment necessity and concerns. PaPA was used in the development of HT&Me to support endocrine therapy adherence.^26–28^

Interviews and focus groups (phases I and II) will draw on elements of PaPA and related models, including Leventhal’s Common Sense Model of Illness and the Necessity/Concerns Framework.^33 34^

Similar to the SWEET programme, SWEET-PLUS will follow MRC and INDEX guidance^35 36^ on intervention development, underpinned by the Person-Based Approach,^37^ to ensure that we prioritise and incorporate user perspectives where possible during the co-development process.

### Phase I

Phase I of the research will involve qualitative interviews and/or focus groups. These will explore experiences of adherence to CDK4/6 inhibitors and identify support needs among women with early-stage ER+ve breast cancer.

### Eligibility and sampling

We will interview women with stage 1–3 ER+ve breast cancer who have been prescribed a CDK 4/6 inhibitor alongside endocrine therapy within the past 18 months; eligibility criteria are summarised in table 2. We will include women who remain on a CDK4/6 inhibitor (irrespective of whether they have had a dose reduction), those with suboptimal implementation (eg, skipping doses, dose reduction), those who have discontinued (e.g personal choice, stopped on clinical advice) and those who have finished treatment. From experience, we anticipate that approximately 25 (and a maximum of 30) interviews will be needed to reach reasonable theoretical sufficiency (conceptual depth)^38^ regarding women’s CDK4/6 inhibitor experiences. The anticipated sample size also aligns with Malterud’s^39^ concept of information power, as SWEET-PLUS has clear research aims, is rooted in established theories and has a specifically niche target population.

**Table 2:** Eligibility criteria for participants in phases I and III

**Inclusion**	**Exclusion**
Women (over 18, no upper age limit) who have early-stage oestrogen sensitive breast cancer and have been prescribed a CDK 4/6 inhibitor alongside endocrine therapy within the past 18 months.	Male patients with breast cancer
Women who remain on a CDK4/6 inhibitor (irrespective of whether they have had a dose reduction), those with suboptimal implementation (eg, skipping doses, dose reduction) and those who have discontinued. They will also be eligible irrespective of whether they had access to a CDK4/6 inhibitor within routine care or as part of a randomised controlled trial.	Women who were initially diagnosed with metastatic breast cancer
Women must have capacity to give informed consent, as judged (where relevant) by their clinical care team.	Women who would need a translator to participate in the study

To ensure that the voices of women from groups with poorer breast cancer outcomes are captured (eg, Black women and those resident in more deprived areas), we will draw on the Centre for Ethnic Health Toolkit^40^ and the INCLUDE ethnicity framework,^41^ and purposively sample potentially eligible patients for ethnic and socio-economic diversity and set quotas (eg, 5–8 Black women and 5–8 women who live in the four most deprived IMD deciles in our sample of ~25). Diversity in other characteristics (eg, age group, CDK4/6 inhibitor drug, other breast cancer treatments) will also be sought across the sample.

### Recruitment

Participants will be recruited through two approaches: NHS hospital sites (primary recruitment route) and non-site routes (supplementary recruitment route).

The three NHS sites are The Newcastle-upon-Tyne Hospitals NHS Foundation Trust, Imperial College NHS Trust and Oxford University Hospitals NHS Trust. To attract women from under-represented backgrounds and communities to participate in the study, we will implement suggestions from our SWEET-PLUS Patient Advisory Group (PAG). We will also work with the research delivery teams at each site to support them to implement the sampling quotas. Strategies to be adopted will include (1) emphasising the importance of diversity in Site Initiation Visits; (2) monitoring recruitment diversity and intervening if necessary; (3) offering practical support around recruitment (eg, providing delivery teams with a look-up table listing postcodes of more deprived areas in the site’s catchment area for use in screening patients for eligibility); (4) signposting delivery staff towards training resources (eg, cultural competency) if they wish to develop their skills in this area and (5) sharing ‘good practice’ (ie, strategies that have worked) across sites.

Supplementary recruitment (non-site) will be conducted through breast cancer support groups and charities with whom we already have close links and social media (eg, Facebook support groups for women on abemaciclib/ribociclib). In our approaches through these networks, we will make it clear that we would like to involve women from different backgrounds. Those interested in taking part will be invited to contact the study team by email, phone or text. Interested individuals will be screened to help ensure diversity. If required, supplementary recruitment will also be conducted via the NIHR’s Be Part of Research Portal (https://bepartofresearch.nihr.ac.uk/).

If required to maximise diversity, we will also use snowball sampling approaches and ask participants to nominate others who fit the eligibility criteria from their networks.

### Data collection

To ensure inclusive research practice,^41 42^ interviews or focus groups with patients will be conducted by telephone, video call or, if the individual strongly prefers (and study resources permit), in person. Additionally, they will be scheduled at a time that suits the participant (eg, avoiding working hours or when they have caring responsibilities) and interviewees will be offered a £25 shopping voucher as recompense for their time.

Interviews and focus groups will follow a semi-structured topic guide (online supplemental material B) informed by past research, team experience and theory (ie, PaPA). Interviews or focus groups will cover experiences of being offered a CDK4/6 inhibitor (including information provision, knowledge and understanding, beliefs about treatment necessity, concerns about the medication and its effects); experiences of taking the medication (including managing it alongside endocrine therapy and any other drugs, side effects, support offered or received with adherence or side effects, dose reductions) and experiences of follow-up care (including regular hospital visits, satisfaction with care) and any unmet needs (eg, information, psychological well-being). The guide will be used flexibly and evolve as the interviews or focus groups progress to ensure that any new issues raised are explored to sufficient depth in subsequent interviews or focus groups.

The same topic guide will be used to inform interview and focus groups, though in the focus groups there will, inevitably, be less opportunity to probe and develop points of discussion. In an attempt to maximise data quality and depth from the focus groups, we have created a code of conduct for participants emphasising the importance of anonymity and confidentiality (online supplemental material C). This will help contribute to a safe environment within which participants can speak freely. Participants will also be sent a follow-up email in case they wish to share any further experiences or follow-up ideas on a 1-to-1 basis. This will provide an opportunity to discuss any personal experiences or disclosures that they might not be comfortable sharing in a group setting.

Participants will also be asked whether they would be willing to be (re-)contacted to participate in later phases of the study (ie, phase III).

### Phase II

Phase II will use a cross-sectional qualitative approach to explore healthcare professionals’ (HCPs’) experience of prescribing or supporting women using CDK4/6 inhibitors alongside endocrine therapy for the management of early breast cancer. We will also explore HCPs’ perceptions of support needs for patients on CDK4/6 inhibitors. This will involve focus groups or interviews, with relevant HCPs involved in prescribing CDK4/6 inhibitors for early breast cancer and/or providing follow-up care.

### Eligibility and sampling

Eligible HCPs will include (but will not be limited to) oncologists, surgeons, cancer nurse specialists, clinical pharmacists and chemotherapy nurses. We will seek diversity among participants in specialty/professional role and years of experience or seniority.

Based on experience, to reach reasonable data sufficiency in relation to participants’ experiences of CDK4/6 inhibitors in early breast cancer, we anticipate recruiting approximately 20 (and up to a maximum of 25) participants. If required, we will run more groups or recruit more interviewees to ensure data sufficiency.

### Recruitment

We will recruit HCPs primarily from the same three NHS hospitals as in phase I, working through the site PIs initially and then through the process of snowballing.

To supplement recruitment, we will (1) advertise the study through Breast Cancer Now’s health professional network which includes 2100 health professionals with an interest in breast cancer and/or (2) conduct focus groups alongside relevant conferences (eg, Association of Breast Surgeons, UK Oncology Nursing Society). We may also recruit through the professional networks of the study team, including Cancer Alliances. These supplementary recruitment routes will provide further diversity of HCP experiences.

### Data collection

Data collection will primarily be through interviews with HCP. We will also offer the option of focus groups (eg, involving two HCP from the same site).

Focus groups or interviews will follow a semi-structured topic guide (online supplemental material D). Topics will include experiences of CDK4/6 inhibitors in early breast cancer; whether/how HCPs advise women about dose reductions and (dis-)continuation; whether/how HCPs judge medication risks and benefits for individual patients; how monitoring for toxicity is undertaken; barriers and facilitators to using the drugs in practice and maintaining adherence; and views on additional support needed. The guide will be used flexibly across focus groups or interviews with new issues raised added to the guide for subsequent focus groups or interviews.

Focus groups or interviews will take place online using Microsoft Teams. They will be scheduled in alignment with HCPs’ schedules. We will seek to run separate groups for medics/consultants and other health professionals so that open discussion is not impeded by professional hierarchies.

### Coding and analysis for phases I and II

Interviews or focus groups will be recorded, transcribed and a framework approach-based thematic analysis will be undertaken.^43^ To develop the analytical framework, the core team (authors PT, LM, EW, LS) will review and discuss the codes for the first two transcripts, ensuring at least two people review each one. Using the framework, one person (PT) will complete coding and analysis, discussing regularly with the core team. Approaches to maintain rigour will be applied (eg, constant comparison, deviant case analysis).^44^ A second stage of analysis will deductively map codes to the elements of PaPA and the supportive care needs framework.^45^ NVivo will be used to support data management.

### Preparations for intervention co-development

Before commencing phase III, findings from phases I and II will be triangulated^46^ to inform the content of the intervention prototype (ie, add-on module to HT&Me). This may include information about how the medication works, the importance of adherence, strategies to support taking it as prescribed, information on key side effects and potential ways to manage these.

An up-to-date review of the literature on CDK4/6 inhibitors in the adjuvant setting will also be undertaken to ensure that information content is evidence-based where relevant. Drawing on Cochrane guidance for rapid literature reviews,^47^ we will specify a tightly focused research question(s) (eg, medication efficacy, risks of toxicity) using the PICO framework. We will work with a medical librarian to develop an efficient search strategy (combining key words and MeSH headings) and search up to three key databases (eg, EMBASE, Medline, CINAHL). Consideration will be restricted to full papers published in English within a defined time frame (eg, since 1 January 2020), reflecting the introduction of CDK4/6 inhibitors in early breast cancer. Screening will be undertaken by a single reviewer, with 10% of citations independently double-screened. Data extraction from relevant studies will be focused and conducted by a single reviewer. A descriptive summary of included studies will be constructed, and narrative synthesis undertaken separately for each research question, if relevant. The review protocol will be made publicly available. Breast cancer clinicians will be on hand to advise throughout, including on interpretation of the evidence.

#### Phase III

Phase III will involve working in partnership with patients with breast cancer to co-develop the intervention content. This will be followed by iterative refinement through user testing. The co-developed and tested content will serve as a prototyped add-on module for the SWEET intervention HT&Me. The existing structure and style of HT&Me will determine some of the design decisions.

### Co-development workshops

We will conduct at least two online workshops to iteratively co-develop intervention content.

Each workshop will have a different group of 5–8 patient participants (ie, women with breast cancer with experience of adjuvant CDK4/6 inhibitors; eligibility criteria as per phase I). Workshop 1 will include women from the PAG, as they have familiarity with the project and objectives and its relationship to the SWEET programme. Workshop 2 will include a mix of women who were interviewed in phase I (and who indicated a willingness to help with later stages of the work) and women who have not previously participated. New participants will be recruited through our three collaborating NHS hospitals (and, if needed, through supplementary non-site routes) with a focus on ensuring diversity. If additional workshops are required to discuss elements of the design or content, recruitment will proceed as for workshop 2.

At workshop 1, we will co-develop an animation storyboard, proposed animation images and draft a script together with participants. Similar to the HT&Me animation,^26^ the co-developed animation will provide information about CDK4/6 inhibitors and address common concerns. This will be followed by discussions about the content participants would like to see in the web-app module. Workshop participants will be provided with access to the HT&Me web-app before the workshop to help inform these discussions. Following the workshop, we will work with the animation company to incorporate feedback and finalise the animation.

Prior to workshop 2, we will work with research software developers to build wire frames (blueprints) of key pages for the web-app. Alongside this, we will develop draft content for the web-app and other relevant content. In designing the web-app and developing content, we will use our learnings from the development of the SWEET programme and expertise of our research software engineer colleagues to ensure that the web-app is easy to access, navigate and uses clear lay language. For intervention content, we will take care to integrate diverse lived experiences (eg, using diverse images of women, quotes from women from different areas, ethnic groups and ages, and review by our diverse PAG) and enhance inclusive design practice.

At workshop 2, the web-app content and wire frames will be reviewed and feedback from participants will be sought. This will be followed by a review of other relevant content. Following the workshops, the prototype will be refined, and the prototype web-app module will be built with the incorporation of the participants’ feedback from the workshop. Similar to phase I, participants in the workshops will receive a £25 shopping voucher as recompense for their time.

### User testing

Iterative user testing of the prototype intervention will involve think-aloud interviews with women in the intervention target population.^48^ A maximum variation sample of women naïve to the intervention design will be recruited; this will comprise a mix of women interviewed in phase I who indicated that they would be willing to be recontacted (and who have not participated in workshop 2) and new recruits. As in phase I, we will work through collaborating hospitals and other non-site routes to ensure ethnic and socio-economic diversity among these new recruits.

Interviewees will view the prototyped animation and web-app module with a researcher present and be invited to say what they are thinking out loud. These interviews will be online using video-conferencing software (unless the participant strongly prefers an in-person interview); we successfully undertook user testing in this way for HT&Me.^26^ They will provide detailed, immediate, observable reactions and inform changes to design and content.

Approximately 10 women will be interviewed in total, in rounds of approximately five (ie, interviews, analysis, refinement, repeat). Interviews will be audio-recorded, transcribed and content analysis conducted. Criteria will be developed for deciding on modifications (eg, likely to impact on adherence, acceptability) and modifications prioritised using the MoSCoW (Must have, Should have, Could have, Would have) criteria.^49^ Pre-testing will cease when no major new issues or concerns are raised in interviews (ie, more than 10 women may be recruited).

### Anticipated outcome

The developed content will serve as a prototype for the add-on module to our existing HT&Me intervention. It will be documented following the TIDieR checklist^49^ with intervention context categorised using the Behavioural Change Taxonomy.^50^ A logic model for change will be developed, aligned where appropriate with the logic model for the HT&Me intervention.^26–28^

Further funding will be sought to test the feasibility and acceptability of the prototype alongside the implementation workstream in our SWEET research programme. Following this, any final changes will be made, and the module will be incorporated into HT&Me.

## Ethics and dissemination

A favourable opinion has been granted from Health Research Authority, Cambridge East Research Ethics Committee (25/EE/0220).

### Consent

Informed consent will be sought before the start of each research encounter. If the interview is taking place remotely, consent will be audio-recorded; the researcher will take the individual through the verbal consent form, and the researcher will sign at the end. Each focus group, co-development and user-testing participant will be consented in the same way before the start of the research encounter. The recordings of consent will constitute a separate record to the interview recording, to enable the separation of personal data and study data. The participant will also be able to ask any further questions they wish at this point of contact with the researcher. Importantly, participants will be informed that their participation is entirely voluntary and that they may withdraw at any time without a reason or any negative consequences.

### Ethical considerations

It is possible that patients participating in qualitative research may have emotional consequences as the interviews may involve them considering and discussing potentially upsetting issues related to their breast cancer. If an interviewee does not wish to answer any question during the interview, this will be respected. This is made clear on the participant information sheet and will be reiterated at the start of the research process. If they become upset, the researcher will ask them if they wish to halt the interview, either temporarily or permanently. If they become very distressed, the researcher will ask whether they would like her to contact someone (eg, family member, friend, GP, consultant, breast cancer nurse) on their behalf. In addition, if taking part in the study has raised questions for the participants about their breast cancer, they will be advised to contact their breast cancer consultant or nurse specialist. Participants with breast cancer will be provided with a debrief sheet, which will also provide signposting to avenues of support.

### Dissemination

The main outputs of the SWEET-PLUS study will be a detailed understanding of (1) the views and experiences of women prescribed CDK4/6 inhibitors (alongside endocrine therapy) for the treatment of early breast cancer and (2) the practices and views of healthcare professionals involved in supporting women on this treatment, leading to (3) the development of an intervention (co-developed with patients) for a prototype ‘add-on module’ to an existing intervention (HT&Me) to support women with adherence to these biological therapies.

Findings will be disseminated via publications in peer-reviewed journals, conference presentations and shared with relevant organisations (eg, UK Breast Cancer Clinical Studies Group, Breast Cancer Now charity). To promote dissemination to patients and lay audiences, our SWEET-PLUS PAG will help us produce lay summaries of the findings which we will host on our study website. We will also enlist the help of members from the SWEET-PLUS PAG and the SWEET Programme PAG with dissemination across their patient networks.

### Patient and public involvement

Women with breast cancer and lived experience of taking CDK4/6 inhibitors have been integral to the development of the study. This has included the establishment of a SWEET-PLUS PAG. PAG members (six women, aged 35–50 with experience of CDK4/6 inhibitors alongside adjuvant endocrine therapy) have reviewed the research questions, grant applications, patient participation information sheets and the phase I interview schedule. The study will continue to be guided by the PAG who will be involved throughout, advising on all phases. This will include activities such as supporting recruitment by identifying and helping address barriers; reviewing and commenting on findings from phases I and II; reviewing the HT&Me website to start identifying core aspects that could be added to support those taking CDK4/6 inhibitors in advance of phase III; assisting with strategies for dissemination of key findings to achieve maximum impact (eg, lay summaries and infographics), etc. Additionally, PAG members will also participate in the first co-development workshop which will start to develop the prototype intervention as part of phase III. Continual engagement with the PAG will ensure the research remains firmly patient-centred and is conducted in a manner that is sensitive and aligned to patients’ needs and experience.

## Discussion

Over the past two decades, there has been an explosion in the availability of oral anticancer drugs, especially costly immunological, biological and precision therapies. Until recently, these were licensed only for use in metastatic disease. In recent years, the approval of CDK4/6 inhibitors (abemaciclib and ribociclib) in the early breast cancer adjuvant setting has led to an increasingly rapid uptake of these medications within the NHS.^24 25^ However, trial data and clinical experience suggest there is significant non-adherence to these drugs,^51^ with many women discontinuing these drugs prematurely. Although some services recognise the challenges of adherence and are seeking to try to address these (eg through monitoring or starting women on lower doses (Chaytor, personal communication, 2025)) as far as we are aware, there are no formal programmes designed to support women with self-management and adherence to these drugs alongside endocrine therapy. SWEET-PLUS will therefore fill an important evidence-gap.

While determinants of non-adherence to abemaciclib and ribociclib are unknown, evidence points to some potentially relevant factors. Treatment regimens for CDK4/6 inhibitors can be complex. Abemaciclib is taken orally in tablet form two times per day and women return to the hospital regularly (every 2 weeks initially, then monthly for a few months) for toxicity monitoring. Ribociclib is taken daily on a 3 weeks on/1 week off cycle, with regular hospital visits (and ECGs) to monitor toxicities. Our related research among patients using biological and precision medicines in advanced cancer highlights the highly stressful nature of regular hospital monitoring.^52^ In addition, multiple side effects can occur with CDK4/6 inhibitors. Up to 20% of patients on abemaciclib may experience diarrhoea, nausea, headaches, infections, anaemia, leucopenia, neutropenia, hepatotoxicity and/or fatigue.^6 53^ Severe diarrhoea, the most common side effect, may be managed with regular loperamide use, and nausea, for which anti-emetics may be helpful. A small proportion of patients experience severe reactions, including pneumonitis or venous thromboembolism.^54^ The most common ribociclib side effects are neutropenia, hepatotoxicity, QT prolongation, joint pain, nausea and headache.^55^ There is a 42% increased risk of venous thromboembolism.^56^ Some severe adverse effects can lead to hospitalisations; others are manageable via dose reductions,^57 58^ but the extent to which dose reductions, or other forms of support, are offered is unknown. As CDK4/6 inhibitors are routinely rolled out within the NHS, the SWEET-PLUS study is well-timed to address this pressing knowledge gap.

## Supplementary material

10.1136/bmjopen-2026-119558 online supplemental file 1

10.1136/bmjopen-2026-119558 online supplemental file 2

10.1136/bmjopen-2026-119558 online supplemental file 3

10.1136/bmjopen-2026-119558 online supplemental file 4

## Data Availability

No data are available.
