# Supplementary material for: Supporting adherence to adjuvant CDK4/6 inhibitors in women with early breast cancer (SWEET-PLUS): protocol for a multicentre UK study using qualitative and co-development methods
Source: BMJ Open. 2026 Aug 11;16(8):e119558. doi: 10.1136/bmjopen-2026-119558 (PMC13475092; doi:10.1136/bmjopen-2026-119558)
Supplement: Supplementary file 1 [file bmjopen-16-8-s001.pdf]

Supplementary Material: A

The **SWEET** (Improving outcome**S** for **W**omen diagnosed with early breast cancer through adh**E**rence to adjuvant **E**ndocrine **T**herapy) research programme is funded by the National Institute for Health Research (NIHR) under the Programme Grants for Applied Research programme [NIHR200098].

Please see page 2 and 3 for an overview of the study and the HT&Me intervention.

You can also find out more about the study here: https://www.sweetstudy.co.uk/

SWEET Programme (NIHR200098)

Underpinnings

- Perceptions and Practicalities Approach (PaPA)
  - Addressing women's beliefs about the necessity of adhering to Adjuvant Endocrine Therapy (AET)
  - Addressing women's concerns about AET
  - Adopting a no-blame approach that is respectful of patient choice, practicalities and priorities
- MRC Guidance

Guiding Principles

- Adopt a person-centred, supportive and tailored approach to ensure the intervention is non-judgemental, accessible and inclusive in both content and visual design
- Support self-management of AET and side-effects through tailored, practical and informational support
- Develop a potentially scalable and implementable intervention within the NHS
- GUIDED and TIDieR guidance

HT&Me Intervention Components

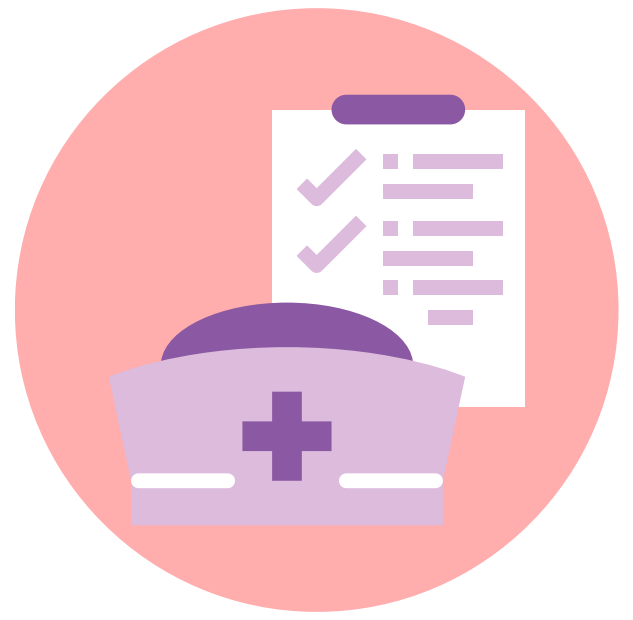

Nurse Consultations

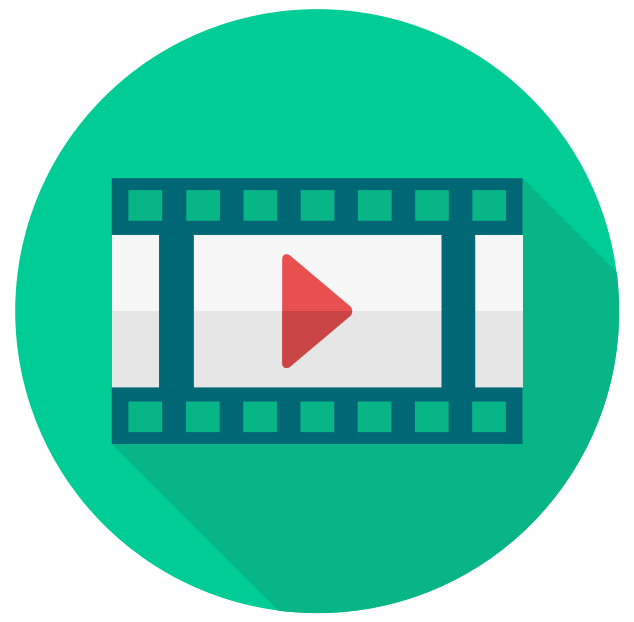

Short Animation

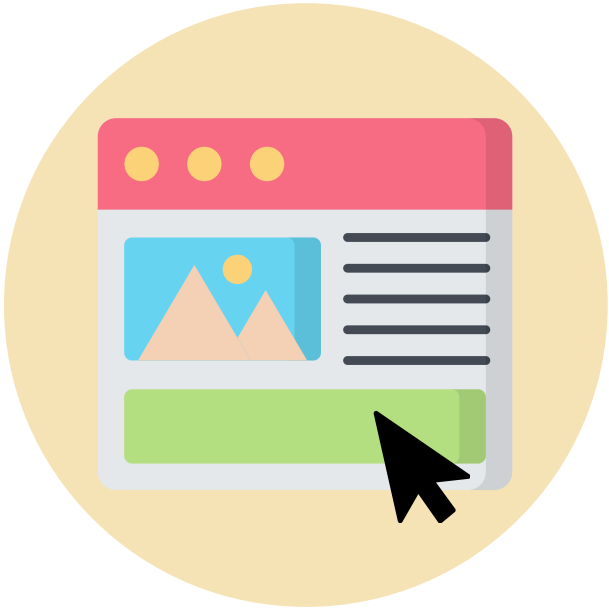

HT&Me Web-app

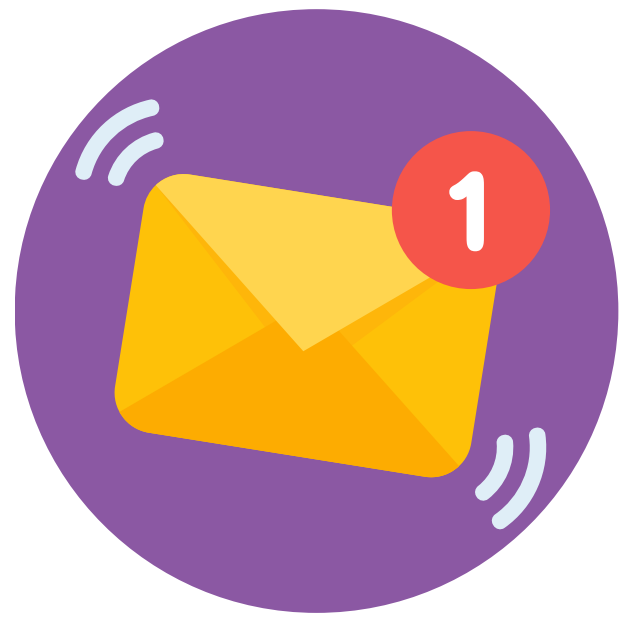

'Nudge' Reminders

Intervention related publications

- Stewart SJ et al. ***Development of the HT&Me intervention to support women with breast cancer to adhere to adjuvant endocrine therapy and improve quality of life.*** Breast. 2023 Aug; 70:32-40. doi:10.1016/j.breast.2023.05.007. 2023 May 27.
- McGeagh L et al. ***Improving outcomeS for Women diagnosed with early breast cancer through adhErence to adjuvant Endocrine Therapy (SWEET): study protocol for a pragmatic randomised control trial of a patient-centred intervention to improve adherence to endocrine therapy in early breast cancer.*** Trials. 2025 Nov 26;26(1):551. doi: 10.1186/s13063-025-09056-6. PMID: 41299751
- McGeagh L et al. ***Supporting women with adherence to adjuvant endocrine therapy (SWEET): feasibility study of the HT&Me intervention.*** Eur J Oncol Nurs. 2026 Feb;80:103026. doi: 10.1016/j.ejon.2025.103026. Epub 2025 Nov 1

HT& ME Intervention

Intervention Components

*AET: Adjuvant Endocrine Therapy

Nurse Consultations

Initial Consultation

- Tailored consultation with SWEET study practitioner (e.g.: breast care nurse) trained to deliver HT&Me
- Consultations are approx. 30 mins and take place in-person/remotely to discuss patient's beliefs, practical barriers and concerns about adherence to AET
- Introduction of the HT&Me web-app
- Facilitate user onboarding and troubleshooting to ensure access

Follow-up Consultation

- Tailored consultation performed after 3 months (approx) to discuss patient's specific concerns, beliefs and practical barriers to AET
- Reinforce importance of AET, refer patient back to relevant HT&Me web-app sections, signposting to additional resources

Short Animation

Short animated video (6 mins)

- Created with healthcare professionals and women who have experience of AET
- Address common concerns and questions about AET (e.g.: how does it work, why are there side effects, etc.)
- Address the importance of AET
- Features women from diverse backgrounds and experiences of hormone therapy to ensure inclusive visual design and content
- Enhance accessibility through lay language, captions and transcripts

Aim:

- Increase knowledge about AET
- Increase necessity beliefs
- Reduce concerns
- Remove practical barriers to taking AET

HT&Me Web-app

Information-based content

Provides information and links to additional sources of support for common concerns and experiences of hormone therapy through the following sections:

- *Taking hormonal therapy*
- *Dealing with side effects*
- *Healthy Living, Healthy Mind*
- *Help & Support*

Interactive sections

My personal support

- Opportunity to input personal beliefs and barriers to AET
- Offers tailored informational support

My Hormone Therapy Diary

- Record side effects and medication intake, set text/email reminders to take AET, ordering and collecting prescriptions

My Goals and Plans

- View, review or set physical or healthy eating goals, plan to take AET, CBT-related activities

'Nudge' Reminders

Regular reminders through messages or email

Women are sent two types of 'nudge' messages via email or text message according to individual preference to:

- prompt adherence, reinforce the importance of continuing therapy, and indicate support is available if needed via the web-app
- encourage women to visit the *My Personal Support* section of the web-app to access tailored information and support
