# Supplementary material for: Supporting adherence to adjuvant CDK4/6 inhibitors in women with early breast cancer (SWEET-PLUS): protocol for a multicentre UK study using qualitative and co-development methods
Source: BMJ Open. 2026 Aug 11;16(8):e119558. doi: 10.1136/bmjopen-2026-119558 (PMC13475092; doi:10.1136/bmjopen-2026-119558)
Supplement: Supplementary file 2 [file bmjopen-16-8-s002.pdf]

Supplementary Material B: Phase 1 Participant Topic Guide

This is a guide to the broad topics that will be covered in the interview - the participant will be free to dictate the direction and content of the interview. The actual questions will be phrased in a way that is clear and understandable to the participant; initial questions will be follow-up with probes to gather more detail. The early interviews and early stages of analyses may identify additional issues which may be useful to follow up in subsequent interviews. Therefore, the topic guide will be used flexibly and will evolve as the study progresses.

1. **Experiences of being offered medication**
   - Can you tell us a bit about when you were first prescribed abemaciclib (Verzenios) / ribociclib (Kisqali) and what you were told at that time?
   - How did you feel about being prescribed this drug? Had you heard of it previously? Did you feel you understood why you were being asked to take it? Did you have any concerns?

2. **Experiences of taking medication**
   - How long have you been taking it for / or did you take it for?
   - How have you found it? How you have found managing it alongside hormone therapy and any other medicines you are on?
   - Sometimes people miss tablets – either forgetting to take them or deciding they don't need or want to take them. Was that something you ever do/did? If yes, can you tell us more about that?
   - Have you had any side effects? If yes, how have you managed these? What support were you offered? If yes, how was that? If no, what might have helped?
   - Have you had any other issues with taking it? If yes, how have you managed these? What support were you offered? If yes, how was that? If no, what might have helped?
   - What (else) would have been/ would be helpful for you?

3. **Experiences of follow-up care**
    - What sort of follow up are you on / have you had – in general for your breast cancer and specifically for abemaciclib/ribociclib?
    - How have you found that – has it met all your needs?
    - Is there anything else that would be helpful for you?
    - Would you find a website useful – which explains how abema/ribo works, and how to cope with side effects, tips on how to take as prescribed etc?
