# Supplementary material for: Supporting adherence to adjuvant CDK4/6 inhibitors in women with early breast cancer (SWEET-PLUS): protocol for a multicentre UK study using qualitative and co-development methods
Source: BMJ Open. 2026 Aug 11;16(8):e119558. doi: 10.1136/bmjopen-2026-119558 (PMC13475092; doi:10.1136/bmjopen-2026-119558)
Supplement: Supplementary file 3 [file bmjopen-16-8-s003.pdf]

Supplementary Material C

Phase 1 Focus group code of conduct

SWEET-PLUS: Supporting adherence to adjuvant CDK4/6 inhibitors in women with early breast cancer

Purpose

To create a respectful, safe, and confidential environment where participants can share openly about their experiences.

Participant Commitments (Ground rules)

- I will arrive on time so the group can start promptly.
- I will participate in the focus group from a quiet, private space.
- I will turn on my camera, as this helps establish a better atmosphere.
- I will wait my turn to speak and not interrupt other participants and group facilitators.
- I will listen to other participants' views without judgment; differing opinions are welcome.
- I will keep all information shared by other participants confidential and will not discuss it outside the group.
- I will not record, screenshot, photograph, or live-post content from the session to protect other participants' privacy.
- I will not share personal or identifying information about others in chat.
- I understand I can decline to answer any questions or discuss any topics that make me uncomfortable.
- I understand that I can take breaks, mute my microphone, or exit the session at any time.

- If I leave the study during or after the focus group session, I understand that the data collected from me to the point of withdrawal will be retained.

Phase 1: Focus group Confidentiality & Code of Conduct Acknowledgment

SWEET-PLUS: Supporting adherence to adjuvant CDK4/6 inhibitors in women with early breast cancer

I have read and understand the Focus Group Code of Conduct (version 1, dated: 05.01.2026).

I agree to:

- Maintain confidentiality of all information shared by participants.
- Not record, photograph, or share session content outside the group.
- Respect anonymity and not attempt to identify other participants.
- Follow the ground rules and facilitator guidance.

Name: ______________________

Signature: ___________________

Date: _______________________
