# Supplementary material for: Supporting adherence to adjuvant CDK4/6 inhibitors in women with early breast cancer (SWEET-PLUS): protocol for a multicentre UK study using qualitative and co-development methods
Source: BMJ Open. 2026 Aug 11;16(8):e119558. doi: 10.1136/bmjopen-2026-119558 (PMC13475092; doi:10.1136/bmjopen-2026-119558)
Supplement: Supplementary file 4 [file bmjopen-16-8-s004.pdf]

Supplementary Material D: Phase 2 HCP Topic Guide

This is a guide to the broad topics that will be covered in the interview - the participant will be free to dictate the direction and content of the interview. The actual questions will be phrased in a way that is clear and understandable to the participant; initial questions will be follow-up with probes to gather more detail. The early interviews and early stages of analyses may identify additional issues which may be useful to follow up in subsequent interviews. Therefore, the topic guide will be used flexibly and will evolve as the study progresses.

- What have been your experiences of the use of CDK4/6 inhibitors in early breast cancer? What is your specific role generally and with respect to these medicines?
- How well do you think patients understand the purpose and benefits of CDK4/6 inhibitors? Are there any common concerns or questions women raise before starting (or early in the course) of treatment? Are there any common misconceptions?
- How is this patient group managed in your centre? Do you run dedicated clinics for these medications? Which HCPs prescribe them? How are women monitored for toxicities? Which HCP are involved in monitoring and follow-up?
- How are individual patients managed eg how are the medication risks/benefits considered? Are there subgroups of patients for whom you would consider these drugs less appropriate? How easy do you think that women find managing these drugs alongside HT and other medicines?
- What is your experience of patient adherence to these drugs? How likely are women to complete the full treatment course as recommended? Are women asked – or do you or your colleagues routinely ask – about adherence (eg skipping doses, stopping for short or longer periods of time)? Are there any particular points in the treatment course when adherence tends to decline? Do patients' emotional reactions to cancer affect their treatment adherence? What role to family, work and social factors play?

- Are there any service level factors that affect patients' abilities to stay on treatment as recommended/complete the treatment course?
- How well do you feel women tolerate this medication? What happens if women are experiencing side effects? What are your strategies for managing side effects or toxicities - do you recommend dose reductions, pausing, stopping etc? How do you feel patients cope with side-effects and how does this impact adherence?
- How confident do you feel supporting this patient group? Do you feel their needs are well met in your centre? Are there any organisational barriers that make it harder to support this patient group? Are there any gaps, or things that could be improved, in the care of these patients?
