# Supplementary material for: Supporting adherence to adjuvant CDK4/6 inhibitors in women with early breast cancer (SWEET-PLUS): protocol for a multicentre UK study using qualitative and co-development methods
Source: BMJ Open. 2026 Aug 11;16(8):e119558. doi: 10.1136/bmjopen-2026-119558 (PMC13475092; doi:10.1136/bmjopen-2026-119558)
Supplement: Supplementary file 5 [file bmjopen-2026-119558.reviewer_comments.pdf]

PEER REVIEW HISTORY

BMJ Open publishes all reviews undertaken for accepted manuscripts. Reviewers are asked to complete a checklist review form and are provided with free text boxes to elaborate on their assessment. These free text comments are reproduced below.

ARTICLE DETAILS

Title (Provisional)

Supporting adherence to adjuvant CDK4/6 inhibitors in women with early breast cancer (SWEET-PLUS): Protocol for a multi-centre UK study using qualitative and co-development methods

Authors

Teow, Pearlin; McGeagh, Lucy; Cain, Henry; Todd, Adam; Rehman, Farah; Brett, Jo; Levitt, Nicky; Turner, Mark; Stewart, Sarah-Jane F.; Hunt, Emma; Terrado, Hazeline; Watson, Eila; Sharp, Linda

---

VERSION 1 - REVIEW

**Reviewer**	**1**
**Name**	**Archer, Stephanie**
**Affiliation**	**Imperial College London, NIHR Imperial Patient Safety Translational Research Centre**
**Date**	**12-May-2026**
**COI**	**None**

Thank you for asking me to review this study protocol. Overall, I enjoyed reading this protocol and think the final study will provide really useful insights. I have made some suggestions below in terms of writing up of the manuscript (intro and method) rather than critiquing the design itself (which I thought was excellent!).

Intro

1. Consider adding the survival statistic for stage I/II patients in the opening paragraph to contextualise the info that follows

2. Consider including a line explaining how ER+ works (i.e. the impact on physiology)

3. Consider moving the information about the drugs into a box to help with readability. It detracts a little bit from your narrative.

4. A reference for non-adherence would be helpful

5. Might talking about the use of risk reducing medications such as tamoxifen also be helpful?

6. An overview of the HT&Me programme when first mentioned would be helpful rather than just having it in the appendix.

Methods

1. Consider removing the in-text reference here as it breaks the flow: “The use of a theoretical framework can enable a comprehensive perspective (22)”

2. Be consistent with co-design/co-development

3. Seems strange to be focusing on stages 1-3 when you only talk about stages 1-2 in the intro. Maybe adjust the intro so this comes as less of a surprise?

4. Consider if the literature on Information Power (Malterud) might be useful in justifying sample size.

5. Consider including a short reflection on the implications of using interviews and focus groups and the impact of different data collection methods on the data content and quality.

6. Might it be worth saying that “the existing structure and style of HT&Me may determine some of the design decisions”.

7. I’m not sure you can “encapsulate diversity”? Consider if this is the right term.

8. Consider whether workshop two really is co-design or if it is consultation or another function on the ladder of co-production.

VERSION 1 - AUTHOR RESPONSE

	Reviewer's comments	Response
1	Consider adding the survival statistic for stage I/II patients in the opening paragraph to contextualise the info that follows	We have added the net survival statistics for Stage I,II and III patients to the introduction.
2	Consider including a line explaining how ER+ works (i.e. the impact on physiology)	We have added a brief explanation about the mechanisms by which endocrine therapy works in women with ER+ve cancers.

**3**	Consider moving the information about the drugs into a box to help with readability. It detracts a little bit from your narrative.	A box has been created to contain information about the drugs.
**4**	A reference for non-adherence would be helpful	We have added references for non-adherence, [15-17]
**5**	Might talking about the use of risk reducing medications such as tamoxifen also be helpful?	We have included additional information on the different types of medication that may be used to reduce the risk of breast cancer recurrence.

**6**	An overview of the HT&Me programme when first mentioned would be helpful rather than just having it in the appendix.	A brief overview has been added to include the main components of the HT&Me support package.
**7**	Consider removing the in-text reference here as it breaks the flow: "The use of a theoretical framework can enable a comprehensive perspective (22)"	We have removed the in-text referencing.
**8**	Be consistent with co-design/co-development	All mention of co-design have been changed to co-development.
**9**	Seems strange to be focusing on stages 1-3 when you only talk about stages 1-2 in the intro. Maybe adjust the intro so this comes as less of a surprise?	Additional information about Stage 3 disease has been added to provide a better introduction.
**10**	Consider if the literature on Information Power (Malterud) might be useful in justifying sample size.	Thank you for the excellent suggestion. We have incorporated Malterud's work on information power to justify the sample size.
**11**	Consider including a short reflection on the implications of using interviews and focus groups and the impact of different data collection methods on the data content and quality.	Thank you for this point. We have added additional information about how we will seek to maximise the quality and depth of data gathered in the focus groups.
**12**	Might it be worth saying that "the existing structure and style of HT&Me may determine some of the design decisions".	We have edited the text accordingly to reflect the impact of HT&Me exisiting design.
**13**	I'm not sure you can "encapsulate diversity"? Consider if this is the right term.	Thank you for pointing this out. This has been changed to 'integrate diverse lived experiences'.

**14**	Consider whether workshop two really is co-design or if it is consultation or another function on the ladder of co-production.	Thank you for this question. HT&Me was co-designed and co-developed in partnership with breast cancer patients and health professionals. Given that the design of the add-on module will be "constrained" by the existing design of HT&Me, we accept that we are not undertaking co-design "from the bottom up" in phase 3 of the study. We have therefore revised the terminology to indicate that we will be co-developing a module that aligns with the existing design of HT&Me. See also our response to point 8.
